# Outcomes of pediatric acute myeloid leukemia treatment in Western Kenya

**DOI:** 10.1002/cnr2.1576

**Published:** 2021-11-22

**Authors:** Romy E. van Weelderen, Festus Njuguna, Kim Klein, Saskia Mostert, Sandra Langat, Terry A. Vik, Gilbert Olbara, Martha Kipng'etich, Gertjan J. L. Kaspers

**Affiliations:** ^1^ Pediatric Oncology Emma Children's Hospital, Amsterdam UMC, Vrije Universiteit Amsterdam Amsterdam The Netherlands; ^2^ Pediatric Oncology Princess Máxima Center for Pediatric Oncology Utrecht The Netherlands; ^3^ Child Health and Pediatrics Moi University/Moi Teaching and Referral Hospital Eldoret Kenya; ^4^ Wilhelmina Children's Hospital/University Medical Center Utrecht Utrecht The Netherlands; ^5^ Pediatrics Indiana University School of Medicine Indianapolis Indiana USA

**Keywords:** Kenya, low‐ and middle‐income countries, pediatric acute myeloid leukemia, sub‐Saharan Africa, survival

## Abstract

**Background:**

Pediatric acute myeloid leukemia (AML) is a challenging disease to treat in low‐ and middle‐income countries (LMICs). Literature suggests that survival in LMICs is poorer compared with survival in high‐income countries (HICs).

**Aims:**

This study evaluates the outcomes of Kenyan children with AML and the impact of sociodemographic and clinical characteristics on outcome.

**Methods and Results:**

A retrospective medical records study was performed at Moi Teaching and Referral Hospital (MTRH) in Eldoret, Kenya, between January 2010 and December 2018. Sociodemographic and clinical characteristics, and treatment outcomes were evaluated. Chemotherapy included two “3 + 7” induction courses with doxorubicin and cytarabine and two “3 + 5” consolidation courses with etoposide and cytarabine. Supportive care included antimicrobial prophylaxis with cotrimoxazole and fluconazole, and blood products, if available. Seventy‐three children with AML were included. The median duration of symptoms before admission at MTRH was 1 month. The median time from admission at MTRH to diagnosis was 6 days and to the start of AML treatment 16 days. Out of the 55 children who were started on chemotherapy, 18 (33%) achieved complete remission, of whom 10 (56%) relapsed. The abandonment rate was 22% and the early death rate was 46%. The 2‐year probabilities of event‐free survival and overall survival were 4% and 7%, respectively. None of the sociodemographic and clinical characteristics were significantly associated with outcome.

**Conclusion:**

Survival of Kenyan children with AML is dismal and considerably lower compared with survival in HICs. Strategies to improve survival should be put in place including better supportive care, optimization of the treatment protocol, and reduction of the abandonment rate and time lag to diagnosis with sooner start of treatment.

## 1 INTRODUCTION

Pediatric acute myeloid leukemia (AML) is a relatively rare leukemia subtype accounting for 15%–20% of the acute leukemias, which represent one‐third of all childhood cancers.^1^ Based on the global estimated incidence of 429 000 new childhood cancer cases each year,^2^ approximately 25 000 children develop AML annually, of whom 20 000 reside in low‐ and middle‐income countries (LMICs).

Over the past decades, pediatric AML treatment protocols have improved substantially in high‐income countries (HICs) resulting in optimized use of antileukemic chemotherapeutics and supportive care measures, more refined risk‐adapted treatment, and the possibility to offer efficacious salvage therapy to refractory and relapsed patients—including hematopoietic stem cell transplantation (SCT).^3^, ^4^, ^5^ These improvements contributed to a substantial increase in survival for children with AML in HICs, where the current 3‐ to 5‐year probabilities of event‐free survival (pEFS) and overall survival (pOS) are 50%–60% and 70%–75%, respectively.^6^, ^7^, ^8^ However, survival in LMICs seems very poor and does not seem to benefit from the same improvements over the past years.^9^

This gap between survival rates in LMICs and HICs has been attributed to many factors including limited access to health care, delay in the patients' diagnosis, comorbidities like malnourishment or malaria, unavailability or intermittent availability of chemotherapeutics, antibiotics, and transfusion products, lack of qualified personnel,^10^ as well as high rates of treatment abandonment,^11^, ^12^ early death (ED),^13^ and treatment‐related mortality.^14^

Compared to HICs, reports on outcome in LMICs are scarce. In order to ultimately improve survival of children with AML in LMICs, assessment and publication of treatment results in these countries is needed first. This study evaluates the outcomes of Kenyan children with AML and the impact of several sociodemographic and clinical characteristics on the outcomes of these patients.

## 2 METHODS

### 2.1 Setting

Kenya is a lower‐middle‐income sub‐Saharan country in East Africa with a population of nearly 54 million people.^15^ In 2015, it was estimated that 36% of the Kenyan population was living below the national poverty line and in that year, Kenya had a gross domestic product and a gross national income per capita of about 64 billion U.S. dollar (USD) and 1290 USD, respectively.^15^

Twenty percent of the Kenyan population has some form of health insurance, of which 80% concerns the government‐owned National Hospital Insurance Fund (NHIF).^16^ The NHIF covers healthcare for the contributor and their family members, which includes inpatient and outpatient cancer treatment. Those in the formal sector pay a monthly income‐dependent contribution, while those in the informal sector pay 500 Kenyan Shillings (KES) per month, which is equivalent to 5 USD.^17^ For patients who do not have NHIF, the costs for AML treatment, including four chemotherapy courses, prophylactic antimicrobials, and intrathecal therapy are about 41 000 KES (380 USD), based on an average of 1.0 m^2^ per patient. Additional costs for a hospital bed, laboratory procedures, and the treatment of complications are not included in this amount.

This study was conducted at the Moi Teaching and Referral Hospital (MTRH) in Eldoret, a town located in Western Kenya. MTRH is a tertiary referral public hospital with a catchment area of roughly 20 million people,^18^ which is almost 40% of the total Kenyan population.^15^ In 2015, the Shoe4Africa Children's Hospital was opened on the MTRH campus. Here, all types of childhood diseases and cancers are treated. It has a 200 bed‐capacity, of which 20 beds are reserved for pediatric oncology patients. Since 2016, there is a ward solely for children with cancer. From hospital records, the annual number of newly diagnosed pediatric oncology patients has increased from approximately 100–244 between 2010 and 2018. Patients share very limited spaces and there are no individual isolation rooms. Chemotherapy and surgery are generally available treatment options, but radiotherapy and SCT are unavailable. Antimicrobials are mostly available, whereas blood products are not consistently available. A multidisciplinary team is present including pediatric oncologists, pediatricians, surgeons, clinical officers, nurses, pharmacists, pathologists, social workers, nutritionists, and child life specialists. Outpatient cancer care is provided as well. Since 2010, a hospital‐based childhood cancer registry exists.

From 2009 onwards, there has been a pediatric oncology‐twinning program between MTRH and the Netherlands, initially with the VU University Medical Center in Amsterdam and currently with the Princess Máxima Center for Pediatric Oncology in Utrecht. MTRH also collaborates with the AMPATH consortium including Riley Hospital for Children and Indiana University, United States of America.

### 2.2 Study design and patients

A retrospective medical records study was conducted between January 2010 and December 2018. Patients aged between 0 and 16 years who were newly diagnosed with AML at MTRH were included. Patients diagnosed with acute promyelocytic leukemia, juvenile myelomonocytic leukemia, myeloid sarcoma, secondary AML, myelodysplastic syndromes, and AML with Down syndrome were excluded. Diagnoses of AML were suspected upon full hemogram and peripheral blood films and mainly confirmed with morphological examination of bone marrow aspirates. The French‐American‐British classification was sporadically used to determine the morphologic subtype. Flow cytometry was available since 2013, but was not accessible to all patients. Cytogenetic studies were unavailable.

### 2.3 Treatment, supportive care, and palliative care

In general, patients were given the option to start treatment or opt for palliative care after being informed of the prognosis and treatment intensity. There were open discussions with the parents about these two options given the high mortalities the medical team was encountering. Table 1 shows the local AML protocol including two “3 + 7” induction courses, two “3 + 5” consolidation courses, and triple intrathecal therapy (Table S1). Hematopoietic SCT was unavailable. Patients received antimicrobial prophylaxis with oral cotrimoxazole three times a week and fluconazole once daily. The blood product supply was unstable. Prior to induction therapy, patients ideally received allopurinol 10 mg/kg three times a day and were hyperhydrated for 24 h with 3000 mL/m^2^.

**TABLE 1:** Treatment schedule of the local protocol for pediatric acute myeloid leukemia at MTRH, a tertiary referral public hospital in Western Kenya

Chemotherapy	Dose
Induction course 1	
Doxorubicin	50 mg/m^2^, 4 h infusion, day 1, 3, and 5
Cytarabine	100 mg/m^2^, 1 h infusion, days 1–7
Triple intrathecal therapy^a^	Doses according to patients' age^b^
Induction course 2	
Doxorubicin	50 mg/m^2^, 4 h infusion, day 1, 3, and 5
Cytarabine	100 mg/m^2^, 1 h infusion, twice daily, days 1–7
Triple intrathecal therapy^a^	Doses according to patients' age^b^
Consolidation course 1	
Etoposide	200 mg/m^2^, 1 h infusion, days 1–3
Cytarabine	100 mg/m^2^, 24 h infusion, days 1–5
Triple intrathecal therapy^a^	Doses according to patients' age^b^
Consolidation course 2	
Etoposide	200 mg/m^2^, 1 h infusion, days 1–3
Cytarabine	100 mg/m^2^, 24 h infusion, days 1–5
Triple intrathecal therapy^a^	Doses according to patients' age^b^

*Note*: The aim was to give chemotherapy every 4 weeks. During the study period, the infusion time of doxorubicin was increased from 1 to 4 h, the dose of etoposide was increased from 100 to 200 mg/m^2^, and the dose of cytarabine was reduced from 200 to 100 mg/m^2^ during consolidation.

Abbreviations: MTRH, Moi Teaching and Referral Hospital.

^a^ Triple intrathecal therapy included methotrexate, hydrocortisone, and cytarabine.

^b^ See Table S1.

Complete remission (CR) status was evaluated prior to consolidation therapy through bone marrow morphological examination. Patients who achieved CR continued with consolidation therapy if they were in a good clinical condition and ideally had platelets >150 × 10^9^/L, a white blood cell count (WBC) of >2.0 × 10^9^/L, and an absolute neutrophil count of >1.0 × 10^9^/L. Sometimes, the protocol was deviated by not awaiting full blood count regeneration. Patients who did not achieve CR did not proceed therapy with curative intent.

### 2.4 Data collection

Names and inpatient numbers of children diagnosed with AML were extracted from the pediatric cancer registry. Their files were obtained from medical record rooms. Sociodemographic and clinical characteristics were extracted using a standardized data collection form. Sociodemographic characteristics included age at diagnosis, sex, distance to MTRH in kilometers, and NHIF status at diagnosis. Clinical characteristics included information on the referral facility (if any), human immunodeficiency virus status, malaria status, symptom duration before MTRH admission, date of diagnosis at MTRH, AML‐specific characteristics (e.g., levels of hemoglobin, WBC, and platelets at diagnosis, French‐American‐British‐type), treatment details, CR status, and (dates of) events. Secondary malignancies were not documented. Follow‐up phone calls were performed in March and September 2020 and January 2021 to supplement missing data due to lost‐to‐follow‐up and to ensure as much as possible that no deaths were missed. For patients of whom only the month and year of birth/death were documented, the day of birth/death was set on the first day of the month of birth/death.

### 2.5 Definitions, response criteria, and statistical analysis

Events included treatment abandonment, induction failure (i.e., lack of initial treatment with curative intent, refractory disease, death prior to the start of treatment, ED, or death >42 days after the start of treatment but prior to CR evaluation), relapse, and death after completion of treatment. Treatment abandonment was defined as failure to either start or continue treatment with curative intent during four or more consecutive weeks.^19^ Lack of initial treatment with curative intent was defined as a palliative care decision immediately after diagnosis due to incurability. ED was defined as death <42 days after the start of treatment. CR was defined as <5% myeloblasts in the bone marrow aspirate with normal hematopoietic elements, full blood count regeneration, and no evidence of localized disease. Refractory disease was defined as failure to achieve CR after two induction courses. Relapse was defined as >5% myeloblasts in the bone marrow aspirate or reappearance of blasts in the peripheral blood, or the development of extramedullary disease after initial CR. Patient delay was defined as the time from onset of symptoms to first admission at MTRH. Diagnosis delay was defined as the time from first admission at MTRH to confirmation of diagnosis. Treatment delay was defined as the time from first admission at MTRH to start of treatment.

The abandonment rate was calculated from the total number of patients. The ED and CR rate were calculated from the number of patients receiving chemotherapy. The relapse rate was calculated only from the number of patients who achieved CR. Time to relapse was calculated from the date of diagnosis to the date of relapse and was subdivided into early relapse (<1 year from initial diagnosis) and late relapse (>1 year from initial diagnosis).^20^ EFS and OS were calculated from the date of diagnosis at MTRH to the date of the first event or death, respectively, or the date of last follow‐up.

In the main EFS analysis, treatment abandonment and induction failure were included as an event at “day 0”. A second EFS analysis was performed with patients who abandoned treatment censored at the time of abandonment.^19^ The Kaplan–Meier method was used to estimate the 2‐year pEFS and pOS. Survival estimates were compared using the log‐rank test. Analyses were performed using SPSS, version 26. Two‐sided *P*‐values of ≤.05 were considered statistically significant.

## 3 RESULTS

### 3.1 Sociodemographic and clinical characteristics

Between 2010 and 2018, 101 patients diagnosed with AML were registered, of whom 20 medical records were missing. Eight patients were excluded because they were diagnosed with acute promyelocytic leukemia (*n* = 4), juvenile myelomonocytic leukemia (*n* = 2), myeloid sarcoma (*n* = 1), or AML with Down syndrome (*n* = 1). Hence, a total of 73 patients were enrolled, of whom 41 (56.2%) were male. The mean age at diagnosis was 8.6 years (*SD*, 3.8) and the median WBC was 37.9 × 10^9^/L (range, 1.2–226.4 × 10^9^/L). Table 2 shows patients' sociodemographic and clinical characteristics. Twenty‐eight patients (38.9%) had NHIF at diagnosis. Fifteen patients were enrolled in NHIF during treatment, which brings the total NHIF enrollment percentage to 59.7% (*n* = 43).

**TABLE 2:** Sociodemographic and clinical characteristics of pediatric patients with AML at MTRH, Eldoret, 2010–2018

Characteristics	Number of patients; *n* (%)
Age at diagnosis (years), *n* = 73	
Mean (*SD*)	8.6 (3.8)
1–2	7 (9.6)
2–5	6 (8.2)
5–10	29 (39.7)
>10	31 (42.5)
Sex, *n* = 73	
Male	41 (56.2)
Female	32 (43.8)
Year of diagnosis, *n* = 73	
2010	3 (4.2)
2011	3 (4.2)
2012	6 (8.5)
2013	14 (19.7)
2014	12 (16.9)
2015	12 (16.9)
2016	5 (7)
2017	8 (11)
2018	10 (13.7)
Distance to MTRH, *n* = 72	
<50 km	12 (16.7)
50–100 km	18 (25)
≥100 km	42 (58.3)
Referred from another health facility, *n* = 72	
No	4 (5.6)
Yes	68 (94.4)
Type of referral health facility, *n* = 68	
Mission hospital	17 (25)
Primary (level 4) public hospital	10 (14.7)
Secondary (level 5) public hospital	32 (47.1)
Tertiary (level 6) public hospital	5 (7.4)
Private facility	4 (5.9)
Symptom duration before first MTRH admission, *n* = 70	
Median (range)	1 (0.1–10)
<1 month	30 (42.9)
≥1 month	40 (57.1)
Test to establish diagnosis, *n* = 68	
Peripheral blood	6 (8.8)
Bone marrow aspirate	29 (42.6)
Peripheral blood and bone marrow aspirate	33 (48.5)
WBC (×10^9^/L), *n* = 58	
Median (range)	37.9 (1.2–226.4)
<100	45 (77.6)
≥100	13 (22.4)
Platelets (×10^9^/L), *n* = 59	
Median (range)	23 (2–218)
Hemoglobin (g/dL), *n* = 63	
Median (range)	4.5 (2.1–11.2)
FAB‐type, *n* = 43	
FAB‐M1	12 (27.9)
FAB‐M2	20 (46.5)
FAB‐M4	5 (11.6)
FAB‐M5	4 (9.3)
FAB‐M6	2 (4.7)
FAB‐M7	0 (0)
HIV positive at diagnosis, *n* = 59	0 (0)
Malaria positive at diagnosis, *n* = 57	2 (3.5)
CNS involvement, *n* = 26	3 (11.5)
NHIF status at diagnosis, *n* = 72	
No NHIF	44 (61.1)
NHIF	28 (38.9)

Abbreviations: AML, acute myeloid leukemia; CNS, central nervous system; FAB, French‐American‐British; HIV, human immunodeficiency virus; MTRH, Moi Teaching and Referral Hospital; NHIF, National Hospital Insurance Fund; WBC, white blood cell count.

The majority of the patients (94.4%) were referred to MTRH from other health facilities, experiencing a median symptom duration of 1 month. Almost half of the referred patients (*n* = 33, 48.5%) were diagnosed at their referral health facility. None of them had received any treatment by the time of admission at MTRH. In most cases (*n* = 42, 58.3%), patients lived more than 100 km from MTRH. AML diagnosis was mainly confirmed with a bone marrow aspirate performed at MTRH. In three of the six patients without bone marrow confirmation, immunophenotyping of peripheral blood was used to confirm the diagnosis in addition to peripheral blood morphology examination. In the other three patients, only peripheral blood morphology examination was used. In total, only 18 patients had immunophenotyping results available.

The median time from first admission at MTRH to confirmation of AML diagnosis was 6 days (range, 0–65 days) and to the start of AML treatment 15.5 days (range, 0–123 days). Reported reasons for the diagnosis and treatment delays included no prompt tests to establish the diagnosis at admission including peripheral blood films and bone marrow aspirates, long processing times of the diagnostic results, unsuccessful or suboptimal bone marrow specimens, initial misdiagnosis with acute lymphoblastic leukemia (ALL; *n* = 4), and in a few cases, parents gave delayed consents for bone marrow aspirates or treatment.

### 3.2 Outcomes

**FIGURE 1:**
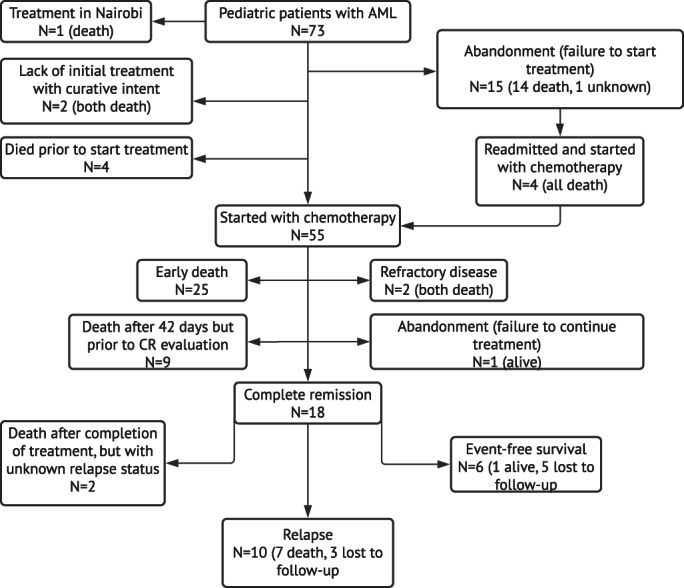
Final outcomes of pediatric patients with AML, MTRH, Eldoret, 2010–2018. AML, acute myeloid leukemia; CR, complete remission; MTRH, Moi Teaching and Referral Hospital

Figure 1 shows the final outcomes of our cohort. One of the 73 patients requested to receive treatment in Nairobi for personal reasons and is therefore not included in the cohort's overview of first events (Table 3) and survival analyses.

**TABLE 3:** First events among pediatric patients with AML at MTRH, Eldoret, 2010–2018

First event		Number of patients, *n*
Treatment abandonment	Failure to start treatment	15
	Failure to continue treatment	1
Induction failure	Lack of initial treatment with curative intent	2
	Refractory disease	1
	Death prior to start treatment	4
	Early death	24
	Death after 42 days but prior to CR evaluation	7
Relapse	Early relapse	7
	Late relapse	3
Death after completion of treatment		2
No event/Event‐free survival		6

*Note*: The patient who requested to receive treatment in Nairobi is excluded from the cohort's overview of first events. The four patients who initially failed to start treatment, but who were readmitted at MTRH and consented to start with chemotherapy are included in the number of patients who failed to start treatment, as this was their first event. Therefore, the number of patients with early death, refractory disease, and death after 42 days but prior to CR evaluation do not exactly correspond with the text and Figure 1.

Abbreviations: AML, acute myeloid leukemia; CR, complete remission; MTRH, Moi Teaching and Referral Hospital.

Two patients were referred to palliative care immediately after diagnosis due to incurability and both died. Sixteen patients (21.9%) abandoned treatment. Of these 16 patients, 15 failed to start treatment after being informed of the prognosis and treatment intensity and one patient failed to continue treatment. Reasons for failure to start treatment were the wish to resort to herbal medications (*n* = 1), financial constraints (*n* = 2), or reasons were not documented (*n* = 12). Four of these patients were readmitted at MTRH and consented to start with chemotherapy 2, 3, or 4 months after initial abandonment. One of these four patients died 1 day after starting treatment. The other three patients completed induction therapy, but two of them died early prior to CR evaluation and one patient died due to refractory disease.

Out of the 55 patients who were started on chemotherapy, including the four patients who initially failed to start treatment but started later, 18 (32.7%) achieved CR, two had refractory disease, and one failed to continue treatment. The patient who failed to continue treatment appeared to have been treated in India and was doing well at time of follow‐up.

The ED rate in our cohort was 45.5% (*n* = 25) and nine patients died >42 days after the start of treatment but prior to CR evaluation. Most ED's occurred ≤14 days after the start of treatment (*n* = 16, 64%). The main causes of ED were respiratory distress (not further specified), excessive hemorrhage, and sepsis, or a combination of these causes.

All patients who achieved CR (*n* = 18) completed therapy, of whom 10 (55.6%) relapsed and two died within 6 months after completing therapy with unknown relapse status. Most relapses (*n* = 7, 70%) occurred early. The median time to relapse was 10.4 months (range, 6.2–18.3 months). None of the relapsed patients received salvage therapy. Six patients are assumed to be in continuous CR with a median follow‐up duration of 5 months (range, 0.3–84.5 months).

Four patients were initially misdiagnosed with ALL and received ALL‐directed treatment for a median duration of 12 days (range, 7–18 days). After their revised diagnosis, they all switched to AML treatment, but died early.

**FIGURE 2:**
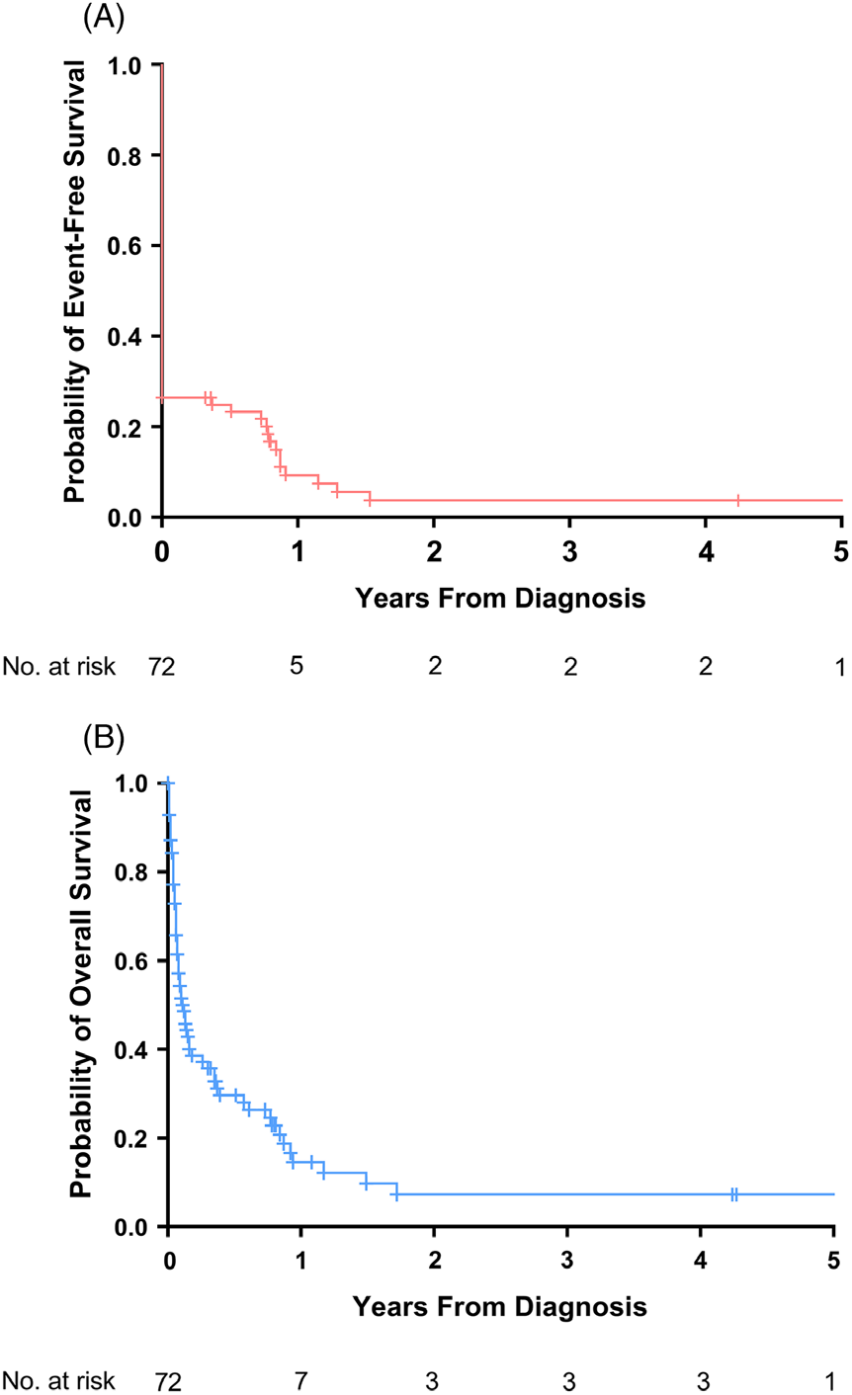
Kaplan–Meier estimates of event‐free survival (A) and overall survival (B) in pediatric patients with acute myeloid leukemia, MTRH, Eldoret, 2010–2018 (*n* = 72)

The 2‐year pEFS and pOS were 3.7% (*SE*, 2.5%) and 7.2% (*SE*, 3.7%), respectively (Figure 2). If patients who abandoned treatment were censored at the time of abandonment, the 2‐year pEFS increased to 6.8% (*SE*, 4.5%; Figure S1). If the one patient who failed to start treatment and the three relapsed patients with unknown statuses at time of follow‐up were assumed to be deceased, the 2‐year pOS dropped to 5.3% (*SE*, 2.9%; Figure S1).

None of the sociodemographic and clinical characteristics (i.e., age, sex, distance to MTRH, WBC at diagnosis, NHIF status at diagnosis) were statistically significant associated with the 2‐year pEFS and pOS (Table 4).

**TABLE 4:** Survival estimates according to sociodemographic and clinical characteristics of pediatric patients with AML, MTRH, Eldoret, 2010–2018

Characteristic	2‐year pEFS		2‐year pOS	
	Percentage (SE)	*P* * (log‐rank)	Percentage (SE)	*P* * (log‐rank)
Age (years)		.17		.64
<10	0 (0)		5.7 (5)	
≥10	7.3 (4.9)		8.7 (5.6)	
Sex		.23		.36
Male	3.3 (3.2)		7.6 (4.9)	
Female	3.9 (3.7)		6.1 (5.6)	
Distance to MTRH		.88		.97
<100 km	6.7 (4.6)		13.3 (6.2)	
≥100 km	0 (0)		0 (0)	
WBC (×10^9^/L)		.83		.88
<100	3 (3)		7 (4.5)	
≥100	7.7 (7.4)		20.8 (12.6)	
NHIF status at diagnosis		.75		.40
No NHIF	3.5 (3.4)		11 (6.4)	
NHIF	3.7 (3.6)		3.7 (3.6)	

Abbreviations: AML, acute myeloid leukemia; MTRH, Moi Teaching and Referral Hospital; NHIF, National Hospital Insurance Fund; pEFS, probability of event‐free survival; pOS, probability of overall survival; SE, standard error; WBC, white blood cell count.

* *P‐*values indicate whether the differences are significant between the subgroups.

## 4 DISCUSSION

This study, which is one of the few to report on pediatric AML treatment outcomes in sub‐Saharan Africa, demonstrates very dismal chances on survival for children with AML in Kenya. The 2‐year pEFS and pOS of 4% and 7%, respectively, are in marked contrast to the current 3‐ to 5‐year pEFS and pOS in HICs that exceed 50% and 70%,^6^, ^7^, ^8^ respectively, as well as to the survival rates reported in other LMICs.^9^ In Mexico and Brazil, two upper‐middle‐income countries, the 5‐year pEFS and pOS were reported to be 33% and 51%, and 31% and 38%, respectively.^21^, ^22^ El Salvador, Morocco, and India, three lower‐middle‐income countries, reported respectively a 5‐year pEFS and pOS of 24% and 27%,^23^ and a 4‐year pEFS and pOS of 30% and 42%,^24^ and 28% and 36%.^25^ However, our survival rates are similar to the reported 1‐year pEFS of 6% in Tanzania, which is, like Kenya, a lower‐middle‐income country in sub‐Saharan Africa.^26^ In the Tanzanian study, all children died within 1 year after diagnosis.^26^

The modification of AML treatment protocols from HICs to local settings in LMICs in order to overcome disproportionate treatment toxicity, while maintaining efficacy, is still an ongoing challenge.^27^ Our AML protocol was intensity‐reduced by using a two‐drug induction regimen and a consolidation regimen without high‐dose cytarabine. Still, our ED rate of 46% was remarkably high compared with the ED rates of about 15%–25% reported in other LMICs^13^, ^24^, ^28^, ^29^, ^30^ and the ED rates below 5% in HICs,^31^, ^32^, ^33^, ^34^ where even more intensive induction regimens are used. The high number of EDs contributed largely to the poor survival and can probably be attributed to the lack of optimal supportive care measures (e.g., unavailability of adequate antibiotics in febrile neutropenic patients, unstable availability of transfusion products, absence of air‐conditioned one‐person isolation rooms, poor hygiene within the wards), as reflected in our main causes of ED.

The CR rate of 33% in our study was evidently lower compared with the current CR rates in HICs exceeding 90%,^7^, ^35^, ^36^ but also when compared with the CR rates in many other LMICs.^9^ However, our CR rate was similar to that in Tanzania.^26^ Other literature from sub‐Saharan Africa is lacking, except for one abstract from Uganda that reported a CR rate of 56% with quite a similar induction regimen.^37^ Their supportive care included additionally ciprofloxacin prophylaxis and anti‐infective measures like mouth and skin wash.^37^ This may imply that an extended supportive care regimen can result in a higher CR rate, as a result of less ED's.

There were no deaths during the consolidation phase. The lack of high‐dose cytarabine in this phase,^4^ together with the intensity‐reduced induction regimen are likely to contribute to the high relapse rate and the evidently higher number of early relapses than late relapses. The latter is in contrast to what has been reported previously in a multinational HIC study.^20^ Furthermore, our relapsed patients were not offered salvage therapy, whereas in HICs, successful salvage therapy contributes largely to the 5‐year pOS of over 70%.^6^, ^7^, ^8^ Currently, intensive re‐induction therapy and hematopoietic SCT are not feasible in Kenya. Lastly, the assessment of remission at the end of induction was based on morphology only without checking for minimal residual disease. Patients who have minimal residual disease, despite having morphological based remission, are likely to experience a relapse.

We hypothesize that other contributing factors to the poor survival in our study include the patient‐, diagnosis‐, and treatment delays. It was beyond the scope of our study to assess the contribution of these various types of delays, however, the patient delay could be explained by the health seeking behavior of Kenyans, which is affected by the high poverty level.^15^ Families seek medical care only when the child is very ill. The low NHIF rate of only 40% among our patients also reflects this. Families without NHIF would therefore have challenges paying for healthcare and may delay visiting health facilities. Nonetheless, NHIF at diagnosis was not significantly associated with better survival, which is in contrast to a previous study among patients with Wilms tumor in the same center.^38^ Considerable travel distances probably contributed to the patient delay as well, however, this was not significantly associated with outcome in our study, like all other sociodemographic and clinical characteristics. The latter is possibly due to the overall poor survival. The diagnosis and treatment delays could be attributed to the inability of healthcare workers to recognize AML, as AML presents quite similarly as other common diseases in sub‐Saharan Africa (e.g., malaria, human immunodeficiency virus, iron deficiency anemia).^39^ There could also be inadequate training on childhood cancer in the training schools. Also, the poor clinical status patients present with and how quickly the parents give consent for diagnostic procedures and treatment may have contributed to the poor survival. The former requires stabilization including getting blood transfusions that are very poorly available in Kenya. The latter is due to the non‐individualistic way of decision‐making of the communities in Kenya. The mother, who is usually with the child, has to get consent from the father who also, at times, needs to talk to extended family members to make the decisions. Furthermore, due to limited personnel, the processing of bone marrow samples frequently takes a long time. Also, the use of morphology alone is limited in terms of making a conclusive diagnosis. Immunophenotyping can aid in the differentiation between ALL and AML subtypes, but it was not routinely done in our study population due to high costs. It was usually only requested if the bone marrow morphology was inconclusive, leading to a delay as well.

Treatment abandonment has been described as an important cause of treatment failure in pediatric AML in LMICs. Our abandonment rate of 22% was similar to the rate of 20% reported in India,^40^ but higher compared to the rate of 4% in Tanzania, where the low abandonment rate was explained by topographic factors and the dedicated oncology team.^26^ In a previous study from MTRH (2007–2009), the abandonment rate of children with cancer was 54%, but the number of children with AML in that cohort was low.^41^ In two other studies performed at MTRH (2010–2012),^38^, ^42^ the abandonment rates of children with Wilms tumor and non‐Hodgkin lymphoma were 31% and 35%, respectively. Lower abandonment in pediatric AML patients than in other pediatric patients at MTRH may be explained by the intensified emphasis on guided assistance in NHIF enrolment at MTRH over the years, thereby effectively lowering the threshold to abandon treatment. Another explanation may be that children with AML are too sick to return home and remain in the hospital until they die. One may argue whether the 12 patients who failed to start treatment and for whom no reasons for not starting treatment were documented should have been classified as abandonment. The medical files of these patients only stated that the parents opted for no treatment after being informed of the prognosis. The medical team's opinion of the curability of these patients was lacking. Although chances are small, survival of pediatric AML is possible in this setting. The fact that it was an open discussion with the parents whether it was wise to start intensive chemotherapy led us to classify these patients as abandonment.

Our study focuses, as one of few, on outcomes of pediatric AML in a sub‐Saharan African country, thereby contributing to more awareness and a better understanding of this disease and its treatment challenges in these countries. Follow‐up was conducted by phone call, whereas lost‐to‐follow‐up often has been a substantial problem in previous studies. The main limitations of this study remain the retrospective design and the relatively small sample size. Data collection from medical records was occasionally hampered by missing or torn pages, or unrecognizable handwriting, and unfortunately, 20 files were completely missing.

In conclusion, outcomes of Kenyan children with AML are dismal and considerably inferior to those in HICs and non‐sub‐Saharan LMICs. Reasons for the poor survival include a lack of adequate supportive care, high relapse rate, high abandonment rate, delays in seeking medical care, in diagnosis of AML, and/or in the start of treatment, and lack of salvage therapy. Future recommendations to improve treatment outcomes in our center include the implementation of a more extended supportive care regimen with the addition of ciprofloxacin prophylaxis in neutropenic patients, a single‐agent etoposide pre‐phase regimen to bridge the time to more intensive induction therapy,^27^ and consolidation therapy including high‐dose cytarabine courses.^27^ Hopefully, this will reduce the number of EDs, increase the CR rate, and lower the relapse rate. Meanwhile, we encourage colleagues working in LMICs to report on their experiences with pediatric AML patients, so that we can identify more successful treatment regimens to further optimize treatment and outcome in LMICs like Kenya.

## CONFLICT OF INTEREST

The authors declare that there is no conflict of interest.

## AUTHOR CONTRIBUTIONS

*Conceptualization*, F.N., G.K., S.M., T.V. and G.O.; *Methodology*, F.N., G.K., S.M., T.V., S.L. and G.O.; *Investigation*, R.W., S.L., and M.K.; *Formal Analysis*, R.W.; *Writing*—*Original Draft*, R.W.; *Writing*—*Review & Editing*, R.W., K.K., S.M., S.L., T.V., G.O., F.N. and G.K.; *Visualization*, R.W.; *Supervision*, K.K., S.M., T.V., G.O., F.N., G.K.; *Project Administration*, R.W.

## ETHICAL STATEMENT

The MTRH's Institutional Research and Ethics Committee approved this study with reference number IREC/2018/103.

## Supporting information


**FIGURE S1** Kaplan–Meier estimates of (A) event‐free survival in pediatric patients with acute myeloid leukemia if patients who abandoned treatment were censored at the time of abandonment and (B) overall survival if the patient who failed to start treatment and the three relapsed patients with unknown statuses at time of follow‐up were assumed to be deceased (*n* = 72).

**TABLE S1** Intrathecal drug dosages according to the patients' age

## Data Availability

The data that support the findings of this study are available from the corresponding author upon reasonable request.
