# Supplementary figures and images for: Outcomes of pediatric acute myeloid leukemia treatment in Western Kenya
Source: Cancer Rep (Hoboken). 2021 Nov 22;5(10):e1576. doi: 10.1002/cnr2.1576 (PMC9575503; doi:10.1002/cnr2.1576)

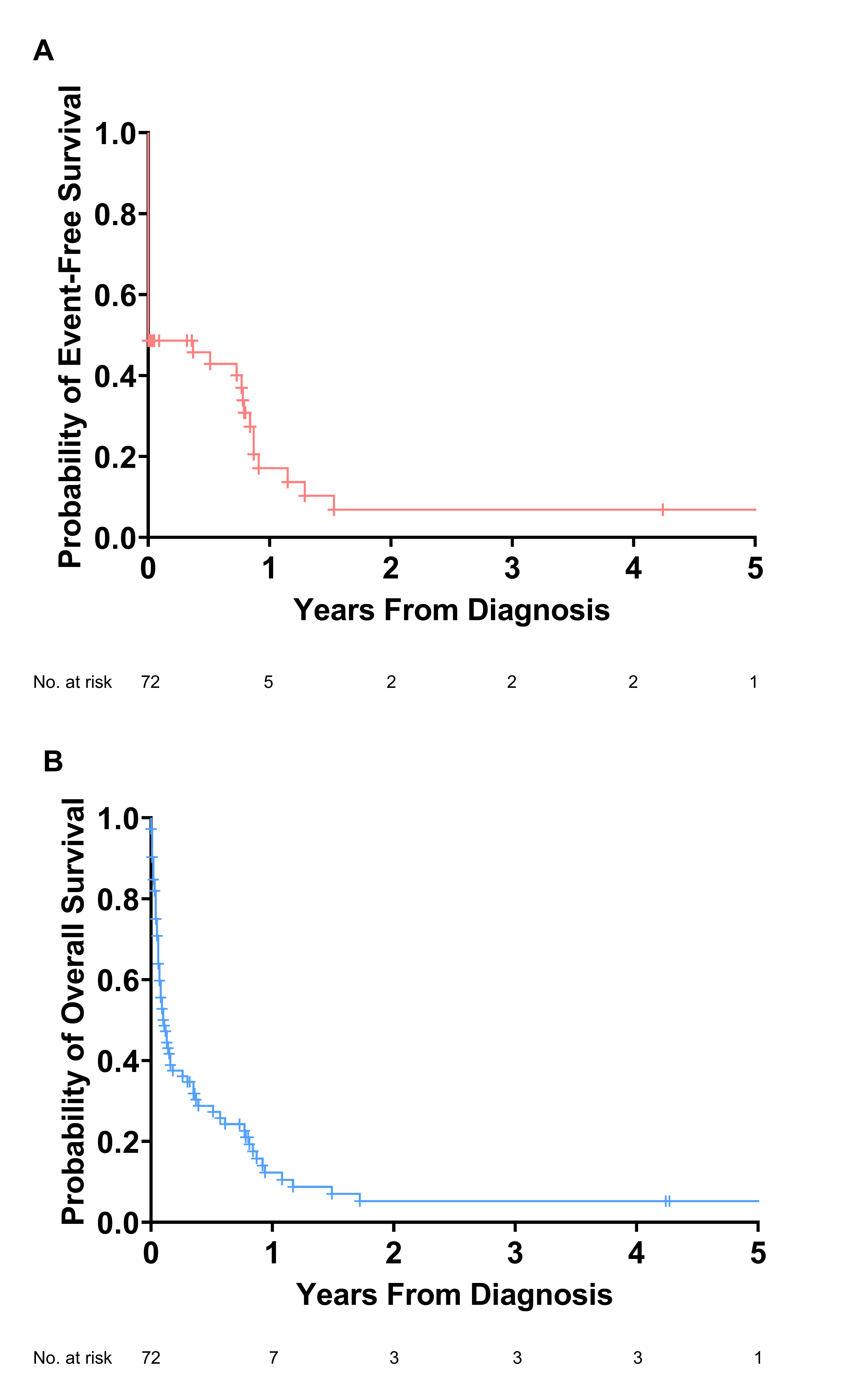

Supplement: Supplementary file 1 — FIGURE S1 Kaplan–Meier estimates of (A) event‐free survival in pediatric patients with acute myeloid leukemia if patients who abandoned treatment were censored at the time of abandonment and (B) overall survival if the patient who failed to start treatment and the three relapsed patients with unknown statuses at time of follow‐up were assumed to be deceased (n = 72). [file CNR2-5-e1576-s001.tif]
