# Supplementary material for: Outcomes of pediatric acute myeloid leukemia treatment in Western Kenya
Source: Cancer Rep (Hoboken). 2021 Nov 22;5(10):e1576. doi: 10.1002/cnr2.1576 (PMC9575503; doi:10.1002/cnr2.1576)
Supplement: Supplementary file 2 — TABLE S1 Intrathecal drug dosages according to the patients' age [file CNR2-5-e1576-s002.docx]

**Supporting Information File**

TABLE S1 Intrathecal drug dosages according to the patients’ age

| Age  Drug | <1 year | 1-2 years | 2-3 years | 3-4 years | ≥4 years |
| --- | --- | --- | --- | --- | --- |
| Methotrexate | 6 mg | 8 mg | 10 mg | 12 mg | 12 mg |
| Cytarabine | 15 mg | 20 mg | 25 mg | 30 mg | 40 mg |
| Hydrocortisone | 6 mg | 8 mg | 10 mg | 12 mg | 12 mg |
